# Characterizing the essential oil composition and assessing the antioxidant and antimicrobial properties of two compositae taxa: *Gerbera delavayi* Franch. and *Gerbera piloselloides* (L.) Cass

**DOI:** 10.3389/fpls.2025.1527525

**Published:** 2025-05-08

**Authors:** Junkai Wu, Wanjun Hu, Jing Chen, Jianping Hu, Cuimin Ke, Zunlai Sheng

**Affiliations:** ^1^ School of Pharmacy, Quanzhou Medical College, Quanzhou, China; ^2^ College of Veterinary Medicine, Northeast Agricultural University, Harbin, China; ^3^ Heilongjiang Key Laboratory for Animal Disease Control and Pharmaceutical Development, Northeast Agricultural University, Harbin, China

**Keywords:** essential oil, *Gerbera delavayi* Franch., *Gerbera piloselloides*, chemical composition, antioxidant activity, antibacterial activity

## Abstract

**Introduction:**

*Gerbera piloselloides* (L.) Cass. and *Gerbera delavayi* Franch. are increasingly recognized for their medicinal properties, particularly among ethnic minority communities in southern China, where they are used for heat-clearing, detoxification, cough relief, lung expulsion, and asthma alleviation. Despite their traditional use, these species have been subjected to limited research regarding their biological activities, leaving a gap in scientific understanding.

**Methods:**

This study was designed to investigate the essential oil (EO) compositions, as well as the antioxidant and antimicrobial properties of *G. piloselloides* and *G. delavayi*. The EOs were extracted via hydrodistillation and analyzed using gas chromatography-mass spectrometry (GC-MS). The antioxidant potential was assessed through ABTS and DPPH free radical scavenging assays, along with the ferric reducing antioxidant power (FRAP) method. The antimicrobial activity was evaluated against five bacterial strains, including two Gram-positive (*Staphylococcus aureus*, *Listeria*) and three Gram-negative (*Salmonella*, *Escherichia coli*, *Pasteurella multocida*) species, using the broth microdilution technique.

**Results:**

The essential oil from *G. piloselloides* (EOgp) yielded 0.14% and was found to contain 24 compounds. It demonstrated high antioxidant activity in the ABTS assay and exhibited the strongest antibacterial effect against *Listeria in vitro*. In contrast, the essential oil from *G. delavayi* (EOgd) had a higher yield of 0.26% and contained a more complex composition with 100 compounds. It showed superior antioxidant activity in both the DPPH and FRAP assays and also demonstrated the highest antibacterial activity against *Listeria*.

**Discussion:**

The findings of this study confirm that both *G. piloselloides* and *G. delavayi* possess significant potential as natural sources of antioxidants and antibacterial agents, warranting further exploration for their development into therapeutic products.

## 1 Introduction

The relentless progression of antibiotic resistance, particularly among multidrug-resistant bacterial strains, poses a significant threat to global health, underscoring the urgent need for the continuous development and discovery of new antimicrobial materials (Baran et al., 2023). While the “Antibiotic Era” may be waning, the potential of medicinal plants as a source for novel antimicrobial agents remains a beacon of hope. Through the intricate process of photosynthesis, plants synthesize a wealth of organic matter and secondary metabolites, which exhibit a broad spectrum of biological activities. These compounds lay the pharmacological groundwork for the prevention and treatment of diseases (Petric et al., 2020; Rehman et al., 2020). With many medicinal plants recognized for their safety, efficacy, and minimal side effects, the exploration of their bioactive compounds for antimicrobial properties is not only imperative but also a promising avenue in the fight against multidrug-resistant bacteria (Bouarab Chibane et al., 2019).

Essential oils (EOs), a type of secondary metabolite produced by aromatic plants, exhibit a spectrum of biological activities, including antibacterial, antioxidant, anti-inflammatory, enzyme inhibitory, sedative, anxiolytic, and antidepressant properties (Zengin et al., 2019; Liu Y. et al., 2024). EOs are utilized as natural remedies for the treatment of infectious diseases and as flavoring agents in food, offering a green and healthy alternative (Coelho et al., 2023; Wu et al., 2024). Due to their remarkable biological activities, EOs from medicinal plants are of great interest to scientists seeking to identify new phytochemical bioactive molecules that align with biodiversity and medicinal needs (Oliveira de Veras et al., 2020).

Gerbera Cass., a member of the Compositae family (Mutisieae Cass.), comprises approximately 80 species ranging from Africa to East Asia, with 20 species found in China, predominantly in the southwestern region (Zhao et al., 2024). *Gerbera piloselloides* (L.) Cass. and *Gerbera delavayi* Franch. are perennial herbs within the Gerbera genus. *G. piloselloides* is known for its heat-clearing, detoxifying, cough-relieving, phlegm-resolving, and circulation-regulating properties (Zhao et al., 2022; Liu C. et al., 2024). Traditionally, it is used in southwestern China to treat cough and sore throat when mixed with honey and also serves as a flavoring agent in winemaking and meat cooking due to its pleasant aroma (Zhou et al., 2022). The plant’s bioactive compounds, including caffeic acid derivatives, parasorboside derivatives, coumarins, and flavonoids, have been isolated through activity-guided isolation (Wang et al., 2014). The EO of *G. piloselloides*, EOgp, has been analyzed by GC-MS and found to contain fatty acids, terpenes, and aromatic compounds (Tang et al., 2003).

*G. delavayi*, found in open areas and forest margins at altitudes of 1800 to 3200 meters, was historically known as “ignited flowers” or “fireweed” due to its leaf’s combustion-supporting properties (Xu et al., 2017). The soft fiber on the back of its leaves is used in hand-weaving (Zheng et al., 2017). Beyond its use in spinning, *G. delavayi* holds significance in medicine and ornamental purposes. Gerbera species in China are noted for their antitussive, antipyretic, hemostatic, circulatory, and anti-inflammatory effects (Wu et al., 2005). The ethanol extract of *G. delavayi* has led to the isolation of two new coumarin compounds, gerdelavins A and B, along with 13 known compounds (Liu et al., 2010). Coumarins, characterized by their benzopyrone core, interact with various enzymes and receptors in organisms through weak bonds, conferring a broad range of medicinal potential, including antibacterial, antitumor, and anticoagulant activities (Balewski et al., 2021; Citarella et al., 2024).

In this context, our study endeavors to delve deeper into the properties of two lesser-studied Gerbera species, *G. piloselloides* and *G. delavayi*. The objective was to assess the EO compositions and to explore their antioxidant and antimicrobial potential. Notably, there is a paucity of literature documenting the biological activities of the EOs from these two plant species. Consequently, this investigation stands as the first comprehensive examination of the biological activities of the extracted EOs from *G. piloselloides* and *G. delavayi*, marking a significant contribution to the existing knowledge base.

## 2 Materials and methods

### 2.1 Plant material

To obtain a comprehensive representation of the chemical profile, the entire plants of *G. piloselloides* and *G. delavayi*, encompassing leaves, stems, roots, and rhizomes, were collected from the Stone Forest region of China. Plant materials from two Gerbera species, were meticulously collected in the Stone Forest region of China. The sampling locations were at elevations of 2316 m (24°81′10.55″ N, 103°30′12.83″ E) for *G. piloselloides* and 1689 m (24°46′27.55″ N, 103°17′18.83″ E) for *G. delavayi*, within Shilin County, Kunming, Yunnan Province, in July 2019. The taxonomic identification of these species was conducted by the Professor Huifeng Sun from Heilongjiang University of Chinese Medicine in Harbin, China.

For posterity and to facilitate future studies, voucher specimens were meticulously archived in the Herbarium of the College of Veterinary Medicine. The voucher numbers assigned to *G. piloselloides* and *G. delavayi* are 2031 and 2032, respectively. Following collection, the herbal materials were subjected to natural drying at room temperature. Subsequently, they were finely pulverized using a grinder and preserved at a refrigerated temperature of 4 °C, awaiting subsequent utilization in experimental procedures.

### 2.2 Extraction of essential oils

The essential oils (EOs) from *G. piloselloides* and *G. delavayi* were extracted using the hydrodistillation method, as described by Semerdjieva et al. (2019). For the extraction process, a precise amount of 100 grams of dried plant material was combined with 1000 mL of distilled water in a flask. The extraction was conducted for a duration of 8 h, commencing once the water reached boiling point. Following extraction, the EOs were separated from the aqueous phase with ethyl ether, dried over anhydrous sodium sulfate, filtered, and then subjected to evaporation of the ethyl ether in an oven at 40 °C for one hour. The resulting EO was transferred to amber vials and stored at -20 °C. The yield percentage (w/w) of the oil was determined based on the initial weight of the plant material used.

### 2.3 GC/MS analysis

The compositional analysis of the EOs was performed using an Agilent Technologies Gas Chromatograph model 7697A, equipped with a triple quadrupole detection system and a split-splitless injection port. The chromatographic separation was achieved on a HP-5MS fused silica capillary column (30 m × 250 μm × 0.25 μm) coupled with an Agilent MS Detector. The column temperature program began at 40 °C for 5 min, followed by an increase to 280 °C at a rate of 10 °C/min. An injection volume of 0.8 μL was used with a split ratio of 1: 20, and helium was employed as the carrier gas at a constant flow rate of 20 mL/min. Mass spectra were acquired at an electron energy of 70 eV, with the ion source temperature set at 250 °C. The mass spectra data were recorded within the mass-to-charge ratio (*m/z*) range of 44-550.

The identification of the EO compounds was accomplished by comparing their retention times and mass spectra with reference data in the NIST mass spectra library. The relative percentage contents of the individual compounds were quantified based on the peak areas in the GC-MS chromatograms, following the methodology described by Thabet et al. (2022).

The identification of the EO compounds was accomplished by comparing their retention times and mass spectra with reference data in the NIST mass spectra library. The retention indices were calculated using the linear retention index method, with a mixture of n-alkanes (C8-C20) at a concentration of 1 μg/mL as the reference compounds. The relative percentage contents of the individual compounds were quantified based on the peak areas in the GC-MS chromatograms, following the methodology described by Thabet et al. (2022).

### 2.4 Estimation of total polyphenolic content

The TPC was quantified using the Folin-Ciocalteu method adapted for a 96-well microplate format (Larrazabal-Fuentes et al., 2019). Initially, the EO sample (500 μg/mL) was combined with 10% (v/v) Folin-Ciocalteu reagent at a ratio of 1: 5 and allowed to stand for 5 min. Subsequently, a sodium carbonate solution was added to the mixture at a volume four times that of the sample and the mixture was shaken for 1 min. After incubation for 1 h at 25 °C, the absorbance was recorded at 765 nm using a microplate reader. A calibration curve was generated using gallic acid dilutions ranging from 0 to 1000 μg/mL. The results were expressed as milligrams of gallic acid equivalents per milliliter of EO.

### 2.5 Evaluation of antioxidant activities

The antioxidant potential of the EOs was assessed using the ferric reducing antioxidant power (FRAP) assay, 2,2’-azinobis-(3-ethylbenzothiazoline-6-sulfonic acid) (ABTS) assay, and 1,1-diphenyl-2-picrylhydrazyl (DPPH) scavenging assay, in conjunction with the determination of the TPC. Vitamin C was employed as the standard reference. The protocols outlined below were adapted for use with 96-well microplates. All assays were conducted in triplicate.

#### 2.5.1 DPPH radical scavenging activity assay

The DPPH radical scavenging capacity of the EOs was evaluated using the methodology of Larrazabal-Fuentes et al. (2019). The percent inhibition (I%) was calculated with the formula: I% = [(Ac - As)/(Ac)] × 100, where Ac is the absorbance of the control and As is the absorbance of the sample. The results were reported as IC_50_ values, representing the concentration of EO (μg/mL) required to inhibit 50% of the DPPH radicals in the solution, determined through linear regression analysis of the percentage of residual DPPH versus sample concentration.

#### 2.5.2 FRAP assay

The FRAP assay was performed as described by Tian et al. (2019). An extract solution (500 μg/mL, 10 μL) was mixed with freshly prepared FRAP solution (70 μL), and the change in absorbance was measured at 593 nm after a 30-min incubation at 37 °C. Standard solutions of FeSO_4_·7H_2_O (0-500 μg/mL) and vitamin C (0-200 μg/mL) were used to construct the calibration curve. FRAP results were expressed as milligrams of vitamin C equivalent per milliliter of EO.

#### 2.5.3 ABTS scavenging activity

The ABTS scavenging activity of the EO was determined following the procedures of Kıvrak (2014). ABTS radical cation (ABTS+) was generated by reacting ABTS (7 mM) with potassium persulfate (2.45 mM) at room temperature in the dark for 16 h. The ABTS+ solution was then diluted with ethanol to achieve an absorbance of 0.700 ± 0.005 at 734 nm. This solution (160 μL) was mixed with 40 μL of EO (0-20000 μg/mL), and the absorbance was measured at 734 nm after a 30-minute incubation at 30 °C. Vitamin C at various concentrations (0-200 μg/mL) served as the reference. The percentage scavenging of ABTS radicals was calculated using the equation from the DPPH assay. The results were expressed as IC_50_ values, calculated based on the linear regression of the percentage of residual ABTS versus sample concentration.

### 2.6 Evaluation of antibacterial activity

#### 2.6.1 Microbial strains employed

The antimicrobial efficacy of the EOs was assessed against a panel of bacterial strains, including *S. aureus* CMCC26003, *Listeria* ATCC 19111, *Salmonella* CVCC541, *E. coli* CVCC10141, and *P. multocida* C48-1. These strains were obtained from the Harbin Institute of Veterinary Medicine (Harbin, Heilongjiang, China).

#### 2.6.2 Determination of minimal inhibitory concentrations

The MICs of the EOs were determined using the broth microdilution method, as outlined in the CLSI protocols M60 (CLSI, 2017) and M100 (CLSI, 2018). The procedure involved preparing a stock solution of EO at 25 mg/mL in a mixture of 20% dimethyl sulfoxide (DMSO) and 80% distilled water. Initially, 100 µL of this stock solution was added to the first well of a 96-well plate, followed by serial two-fold dilutions to achieve concentrations ranging from 25 to 0.05 mg/mL (Cui et al., 2018). Subsequently, each bacterial strain was inoculated into LB broth to achieve a McFarland standard of 0.5, diluted 100-fold, and then added to the wells at a volume of 100 µL per well. The MIC was defined as the lowest concentration of EO that inhibited visible growth of the bacterial strains after an incubation period of 16–18 h at 37 °C. Chloramphenicol, at concentrations ranging from 10 to 0.04 mg/mL, was used as a positive control, while a solution of 20% DMSO-80% distilled water served as the negative control. All experiments were conducted in triplicate to ensure accuracy and reproducibility.

### 2.7 Statistical analysis

Significance was determined at a p-value threshold of < 0.05. Data were processed using GraphPad Prism^®^ version 7.0 and presented as mean ± standard deviation (SD). To ascertain statistically significant differences among the groups, a one-way analysis of variance (ANOVA) was performed.

## 3 Results and discussion

### 3.1 Chemical composition of essential oils

**Figure 1:**
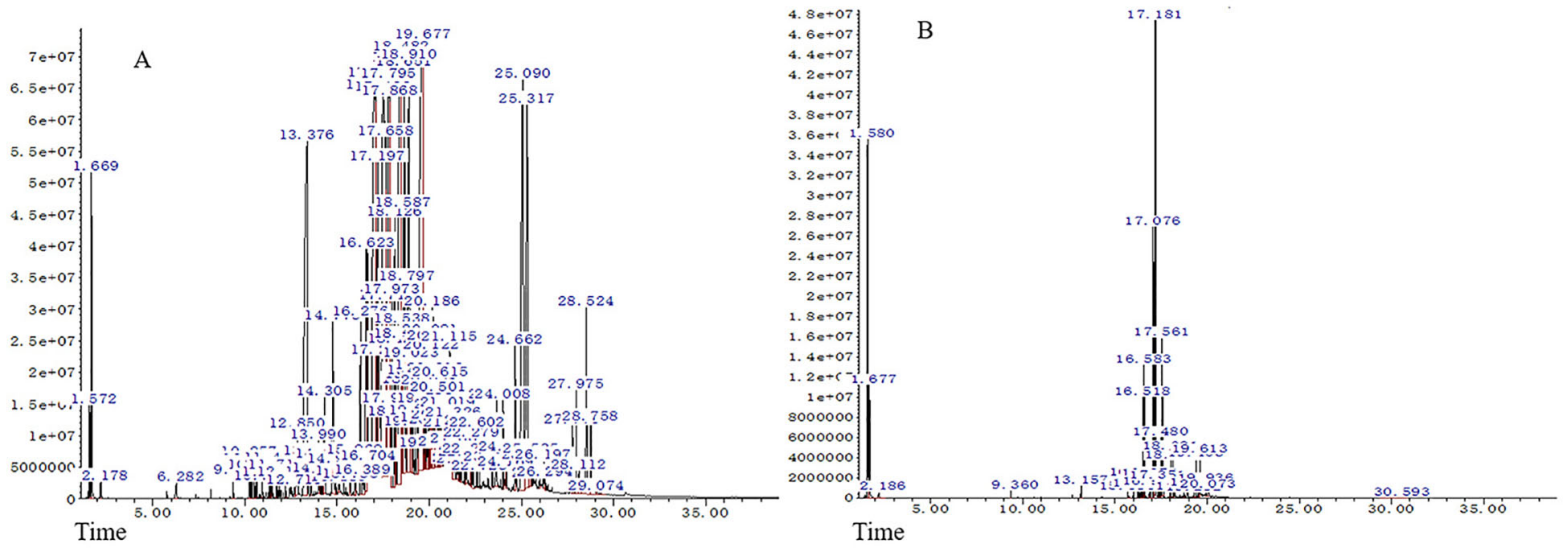
GC/MS profiles of the essential oils from *Gerbera piloselloides* **(A)** and *Gerbera delavayi* **(B)**.

The medicinal parts of two Gerbera species, namely the whole plants of *G. piloselloides* and *G. delavayi*, were subjected to hydrodistillation, yielding yellow EOs with distinctive odors. The yields for *G. piloselloides* and *G. delavayi* were 0.14% (w/w) and 0.26% (w/w), respectively. The chemical compositions of these EOs were elucidated using GC/MS. The compositional percentages of the EOs from *G. piloselloides* and *G. delavayi* are presented in **Tables 1** and **2**, respectively. The total ion chromatograms for the EOs of both species (EOgp and EOgd) are depicted in **Figure 1**. GC/MS analysis of EOgp identified 24 components, with berkheyaradulene (32.03%), 4-(2’, 4’, 4’-trimethyl-cyclo[4.1.0]hept-2’-en-3’-yl)-3-buten-2-one (12.86%), caryophyllene (6.78%), and cycloisolongifolene (5.30%) as the principal constituents. In contrast, EOgd comprised 100 components, with butanoic acid, 3,7-dimethyl-2,6-octadienyl ester, (*E*)- (10.50%), cyperene (9.70%), β-panasinsene (7.13%), benzamide, *N*-(1-adamantyl)-2-hydroxy- (6.12%), and benzene, 1-(1,1-dimethylethyl)-4-ethyl- (5.31%) as the predominant compounds.

**Table 1:** Constituents of the essential oil from *Gerbera piloselloides.*.

No.^a^	Components	Retention time (min)	RI^b^	CAS number	Molecular formula
1	Ethanol	1.58	61	64-17-5	C_2_H_6_O
2	Ethyl ether	1.677	73	60-29-7	C_4_H_10_O
3	Ethyl acetate	2.186	136	141-78-6	C_4_H_8_O_2_
4	(+)-alpha-Pinene	9.36	1024	7785-70-8	C_10_H_16_
5	Adipic acid, di(trans-hex-3-enyl) ester	13.157	1494	No	C_18_H_30_O_4_
6	Thymol	15.686	1807	89-83-8	C_10_H_14_O
7	1-Ethyl-3-(propen-1-yl)adamantane	16.244	1876	No	C_15_H_24_
8	(-)-Aristolene	16.413	1897	6831-16-9	C_15_H_24_
9	(-)-alpha-Gurjunene	16.518	1910	489-40-7	C_15_H_24_
10	Cycloisolongifolene	16.583	1918	No	C_15_H_24_
11	cyclosativene	16.874	1954	22469-52-9	C_15_H_24_
12	alpha-longipinene	16.93	1961	5989-08-2	C_15_H_24_
13	4-(2’, 4’, 4’-trimethyl-yciclo[4.1.0]hept-2’-en-3’-yl)-3-buten-2-one	17.076	1979	No	C_14_H_20_O
14	Berkheyaradulene	17.181	1992	65372-78-3	C_15_H_24_
15	Cyperene	17.351	2013	2387-78-2	C_15_H_24_
16	Longifolene	17.48	2029	61262-67-7	C_15_H_24_
17	Caryophyllene	17.561	2039	87-44-5	C_15_H_24_
18	Humulene	18.021	2096	26259-79-0	C_15_H_24_
19	Carvacryl acetate	18.15	2112	6380-28-5	C_12_H_16_O_2_
20	(+)-DELTA-CADINENE	18.756	2187	483-76-1	C_15_H_24_
21	2,3,5,6-tetramethyl-Phenol	19.282	2252	527-35-5	C_10_H_14_O
22	Neryl 2-methylbutanoate	19.403	2267	51117-19-2	C_15_H_26_O_2_
23	Caryophyllenyl alcohol	19.532	2283	No	C_15_H_26_O
24	Caryophyllene oxide	19.613	2293	1139-30-6	C_15_H_24_O
25	humulene epoxide ii	19.936	2333	19888-34-7	C_15_H_24_O
26	Isocaryophillene	20.073	2350	13877-93-5	C_15_H_24_
	1,1,1,3,5,5,5-Heptamethyltrisiloxane				
27	Total Identified	30.593	3652	1873-88-7	C_7_H_22_O_2_Si_3_
	Monoterpenes			76.61	
	Sesquiterpenes			0.31	
	Phenolic Compounds			55.50	
	Aromatic Compounds			2.73	
	Esters			13.75	
	Other Compounds			1.98	

a Compounds listed in order of elution from the Rtx-5MS capillary column.

b Retention indices relative to C_8_-C_20_ n-alkanes on an Rtx-5MS capillary column.

**Table 2:** Constituents of the essential oil from *Gerbera delavayi*.

No.^a^	Components	Retention time (min)	RI^b^	CAS number	Molecular formula
1	Ethanol	1.572	60	64-17-5	C_2_H_6_O
2	Ethyl ether	1.669	72	60-29-7	C_4_H_10_O
3	Ethyl Acetate	2.178	135	141-78-6	C_4_H_8_O_2_
4	2-Isopropoxyethanol	6.282	643	109-59-1	C_5_H_12_O_2_
5	3-Ethylidenecycloheptene	9.36	1024	–	C_9_H_14_
6	Sabinene	10.257	1135	3387-41-5	C_10_H_16_
7	1-(4-Methylphenyl)ethanol	10.354	1147	536-50-5	C_9_H_12_O
8	6-methyl-5-Hepten-2-one	10.507	1166	110-93-0	C_8_H_14_O
9	2,2-dimethyl-3-octyne	10.62	1180	19482-57-6	C_10_H_18_
10	Alpha -Phellandrene	10.944	1220	99-83-2	C_10_H_16_
11	O-Cymene	11.307	1265	527-84-4	C_10_H_14_
12	D-Limonene	11.404	1277	5989-27-5	C_10_H_16_
13	Eucalyptol	11.469	1285	470-82-6	C_10_H_18_O
14	Beta-Ocimene	11.719	1316	13877-91-3	C_10_H_16_
15	(Z)-linalool oxide (furanoid)	12.196	1375	5989-33-3	C_10_H_18_O_2_
16	(+)-2-Carene	12.713	1439	–	C_10_H_16_
17	cyclene	12.85	1456	508-32-7	C_10_H_16_
18	N-methyl-2-pyrolidene	13.376	1521	33838-11-8	C_5_H_9_N
19	nerol oxide	13.602	1549	1786-08-9	C_10_H_16_O
20	5-methyl-3-(1-methylethylidene)-1,4-Hexadiene	13.99	1597	–	C_10_H_16_
21	(-)-Terpinen-4-ol	14.086	1609	20126-76-5	C_10_H_18_O
22	3-methylene-1,5,5-trimethyl-cyclohexene	14.256	1630	16609-28-2	C_10_H_16_
23	2-hydroxy-4-methylbenzaldehyde	14.305	1636	698-27-1	C_8_H_8_O_2_
24	3-Carene	14.773	1694	13466-78-9	C_10_H_16_
25	2-isopropyl-4-methyl anisole	14.927	1713	31574-44-4	C_11_H_16_O
26	Citral	15.347	1765	5392-40-5	C_10_H_16_O
27	cis-Thujopsene	15.735	1813	470-40-6	C_15_H_24_
28	1-(4-Hydroxy-3-methylphenyl)ethanone	15.969	1842	876-02-8	C_9_H_10_O_2_
29	1,5-dimethyl-2,4-bis(1-methylethyl)-benzene	16.276	1880	5186-68-5	C_14_H_22_
30	1-Isopropyl-4,7-dimethyl-1,2,4a,5,8,8a-hexahydronaphthalene	16.389	1894	5951-61-1	C_15_H_24_
31	(-)-Aristolene	16.583	1918	6831-16-9	C_15_H_24_
32	Cedrene-V6	16.623	1923	–	C_15_H_24_
33	Aciphyllene	16.704	1933	–	C_15_H_24_
34	cyclosativene	17.011	1971	22469-52-9	C_15_H_24_
35	alfa.-Copaene	17.116	1984	138874-68-7	C_15_H_24_
36	4-(2’, 4’, 4’-trimethyl-yciclo[4.1.0]hept-2’-en-3’-yl)-3-buten-2-one	17.197	1994	–	C_14_H_20_O
37	Berkheyaradulene	17.254	2001	65372-78-3	C_15_H_24_
38	(-)-Cyperene	17.496	2031	2387-78-2	C_15_H_24_
39	Caryophyllene	17.714	2058	87-44-5	C_15_H_24_
40	1-(1,1-dimethylethyl)-4-ethyl-benzene	17.795	2068	7364-19-4	C_12_H_18_
41	2,4-diethyl-7,7-dimethylcyclohepta-1,3,5-triene	17.868	2077	–	C_13_H_20_
42	2,5-Dimethylchroman-4-one	17.9	2081	69687-87-2	C_11_H_12_O_2_
43	beta-maaliene	17.973	2090	489-29-2	C_15_H_24_
44	Humulene	18.126	2109	6753-98-6	C_15_H_24_
45	Alloaromadendrene	18.175	2115	25246-27-9	C_15_H_24_
46	rotundene	18.207	2119	–	C_15_H_24_
47	beta-Panasinsene	18.433	2147	56684-97-0	C_15_H_24_
48	Selina-3,7(11)-diene	18.482	2153	6813-21-4	C_15_H_24_
49	gamma-selinene	18.506	2156	515-17-3	C_15_H_24_
50	beta-Guaiene	18.538	2160	88-84-6	C_15_H_24_
51	Alloaromadendrene	18.587	2166	25246-27-9	C_15_H_24_
52	alpha.-Muurolene	18.651	2174	31983-22-9	C_15_H_24_
53	(+)-Calarene	18.797	2192	17334-55-3	C_15_H_24_
54	(+)-DELTA-CADINENE	18.91	2206	483-76-1	C_15_H_24_
55	Guaia-9,11-diene	18.974	2214	–	C_15_H_24_
56	Isolongifolene	19.023	2220	1135-66-6	C_15_H_24_
57	(+)-α-murolene	19.071	2226	17627-24-6	C_15_H_24_
58	1, 1, 5-Trimethyl-1, 2-dihydronaphthalene	19.128	2233	–	C_13_H_16_
59	3-Methyl-2-butenoic acid, 4-methoxybenzyl ester	19.241	2247	–	C_13_H_16_O_3_
60	(E)-3,7-Dimethylocta-2,6-dienyl ethyl carbonate	19.338	2259	–	C_13_H_22_O_3_
61	[(2E)-3,7-dimethylocta-2,6-dienyl] butanoate	19.629	2295	106-29-6	C_14_H_24_O_2_
62	Caparratriene	19.694	2303	–	C_15_H_26_
63	1,3-dimethyl-5-ethylbenzene	19.782	2314	934-74-7	C_10_H_14_
64	.beta.-Guaiene	19.895	2328	88-84-6	C_15_H_24_
65	Delta-Selinene	19.968	2337	473-14-3	C_15_H_24_
66	Alloaromadendrene	20.025	2344	025246-27-9	C_15_H_24_
67	Alpha-Elemene	20.081	2351	5951-67-7	C_15_H_24_
68	dehydro-aromadendrene	20.122	2356	–	C_15_H_22_
69	Xanthurenic acid	20.186	2364	59-00-7	C_10_H_7_NO_4_
70	4,8a-dimethyl-6-prop-1-en-2-yl-1,3,5,6,7,8-hexahydronaphthalen-2-one	20.316	2380	–	C_15_H_22_O
71	1,2,3,5,6,7,8,8a-octahydro-1-methyl-6-methylene-4-(1methylethyl)naphthalene	20.356	2385	150320-52-8	C_15_H_24_
72	4-(2,3,4,6-Tetramethylphenyl)-3-buten-2-one	20.429	2394	–	C_14_H_18_O
73	Epizonarene	20.501	2403	41702-63-0	C_15_H_24_
74	Longifolene	20.566	2411	475-20-7	C_15_H_24_
75	Cedren-13-ol, 8-	20.615	2417	18319-35-2	C_15_H_24_O
76	1,2,3a,6-Tetramethyloctahydrocyclopenta[c]pentalen-3(3ah)-one	20.728	2431	–	C_15_H_24_O
77	7R,8R-8-Hydroxy-4-isopropylidene-7methylbicyclo[5.3.1]undec-1-ene	20.946	2458	–	C_15_H_24_O
78	3,5,6,7,8,8a-hexahydro-4,8a-dimethyl-6-(1-methylethenyl)-2 Naphthalenone	21.019	2467	–	C_15_H_22_O
79	Longipinocarvone	21.229	2493	–	C_15_H_22_O
80	Cadina-1(10),6,8-triene	21.326	2505	1460-96-4	C_15_H_22_
81	2,2,7,7-tetramethyltricyclo[6.2.1.01,6]undec-5-en-4-one	21.6	2539	23747-14-0	C_15_H_22_O
82	6-Isopropenyl-4,8a-dimethyl-1,2,3,5,6,7,8,8a-octahydronaphthalene-2,3-diol	21.721	2554	–	C_15_H_24_O_2_
83	Corymbolone	21.923	2579	97094-19-4	C_15_H_24_O_2_
84	2,2,7,7-tetramethyltricyclo[6.2.1.01,6]undec-5-en-4-one	22.02	2591	23747-14-0	C_15_H_22_O
85	Fukinanolid	22.247	2619	19906-72-0	C_15_H_22_O_2_
86	(2-hydroxy-5-methylphenyl)-(4-methoxyphenyl)methanone	22.279	2623	–	C_15_H_14_O_3_
87	6,10,14-trimethylpentadecan-2-one	22.36	2633	502-69-2	C_18_H_36_O
88	(2-Hydroxy-5-methylphenyl)(4-methoxyphenyl)methanone	22.602	2663	–	C_15_H_14_O_3_
89	1-Methyl-1-silolanyl heptanoate	22.723	2678	–	C_12_H_24_O_2_Si
90	2,6-ditert-butylnaphthalene	23.418	2764	3905-64-4	C_18_H_24_
91	n-Hexadecanoic acid	23.644	2792	57-10-3	C_16_H_32_O_2_
92	N-Mesitytricyclo-[3.2.1.0(2.4)]octane-3-carboxamide	24.008	2837	342394-51-8	C_18_H_23_NO
93	Kaur-15-ene	24.169	2857	5947-50-2	C_9_H_12_N_2_O_5_S
94	2-(4-methoxybenzoyl)-1,6-dimethyl-1,2,3,4-tetrahydropyrrolo[1,2-a]pyrazine	24.202	2861	–	C_17_H_20_N_2_O_2_
95	Kaur-16-ene	24.662	2918	562-28-7	C_20_H_32_
96	N-(1-adamantyl)-2-hydroxybenzamide	25.09	2971	3728-06-1	C_17_H_21_NO_2_
97	2,5-Diethylpyrazine	25.317	2999	013238-84-1	C_8_H_12_N_2_
98	propoxy(dipropyl)phosphane	25.454	3016	6418-60-6	C_9_H_21_OP
99	o-Terphenyl	25.535	3026	84-15-1	C_18_H_14_
100	N-1-Adamantyl-p-nitrobenzalimine	25.987	3082	–	C_17_H_20_N_2_O_2_
101	3-Adamantan-1-yl-3-oxo-propionitrile	26.197	3108	–	C_13_H_14_NO
102	Hexestrol	26.294	3120	84-16-2	C_18_H_22_O_2_
103	1-(4-Methoxyphenyl)-4,6-dimethyl-2(1H)-pyrimidinone	27.773	3303	74360-11-5	C_13_H_14_N_2_O_2_
104	6,6-Diphenylfulvene	27.975	3328	2175-90-8	C_18_H_14_
105	Triphenylene	28.112	3345	217-59-4	C_18_H_12_
106	Benz[a]anthracene	28.524	3396	56-55-3	C_18_H_12_
107	dimethylphenylsilane	28.758	3425	766-77-8	C_8_H_11_Si
108	2,6-dimethylocta-2,4,6-triene	29.074	3464	673-84-7	C_10_H_16_
	Total identified			95.06	

a Compounds listed in order of elution from the Rtx-5MS capillary column.

b Retention indices relative to C_8_-C_20_ n-alkanes on an Rtx-5MS capillary column.

In a prior phytochemical study, the researchers documented the presence of 17 volatile organic components in *G. piloselloides*, encompassing fatty acids, terpenes, and aromatic compounds. Notably, neryl (*S*) -2-methylbutanoate (35.99%), 4-hydroxy-3-methylacetophenone (8.74%), and n-hexadecanoic acid (7.48%) emerged as the predominant constituents of the plant’s essential oil (Luo et al., 2013). Previous studies have identified various volatile organic compounds in specific parts of *G. piloselloides*, such as leaves, caudices, and roots (Tang et al., 2003). However, our study focused on the essential oils extracted from the whole plants of *G. piloselloides* and *G. delavayi*. The observed discrepancies in the identified compounds may be attributed to the varying environmental conditions of the plant collection sites and the inclusion of multiple plant parts in our analysis.

Previous research on Gerbera has revealed the presence of coumarins, sesquiterpenoids, triterpenoids, and cyanogenic glycosides (Liu et al., 2010). Despite the distinct EO profiles of the two Gerbera species, eight compounds, including (-)-aristolene, 4-(2’, 4’, 4’-trimethyl-cyclo[4.1.0]hept-2’-en-3’-yl)-3-buten-2-one, berkheyaradulene, cyclosativene, cyperene, naphthalene, 1,2,3,5,6,8a-hexahydro-4,7-dimethyl-1-(1-methylethyl)-, (1*S*-*cis*)-, humulene, and caryophyllene, are common to both, as detailed in **Table 3**.

**Table 3:** Common chemical compounds in essential oils of *Gerbera piloselloides* and *Gerbera delavayi*.

Compound	Content (%)	
	*Gerbera piloselloides*	*Gerbera delavayi*
(-)-Aristolene	0.357	1.611
cyclosativene	0.753	7.831
4-(2’, 4’, 4’-trimethyl-yciclo[4.1.0]hept-2’-en-3’-yl)-3-buten-2-one	12.862	1.715
Berkheyaradulene	32.025	1.224
Cyperene	0.701	9.700
Naphthalene, 1,2,3,5,6,8a-hexahydro-4,7-dimethyl-1-(1-methylethyl)-, (1S-cis)-	0.268	3.654
Humulene	1.761	1.284
Caryophyllene	6.782	0.77
Total (%)	55.509	27.789

Berkheyaradulene is particularly abundant in *G. piloselloides* (10.50%), representing a sesquiterpene hydrocarbon with an unusual carbon skeleton characterized by a bridgehead carbon connected to three rings, also found in other Asteraceae plants (Szöke et al., 2004). Caryophyllene, notable for its cyclobutane ring, a rare occurrence in nature, is often accompanied by isocaryophyllene and α-humulene, its ring-opened isomer (Taherpour et al., 2010). Cyperene, a tetracyclic sesquiterpene, possesses unique properties such as sterilizing, antioxidant, anticarcinogenic, and immune-boosting functions (Skała et al., 2016; Hu et al., 2017). Thymol, with its thyme oil-like aroma, may contribute to the use of *G. piloselloides* in winemaking and meat cooking. Thymol’s expectorant properties have been documented, and it also exhibits bactericidal effects, suggesting its potential in treating bronchitis and whooping cough (Zhou et al., 2019). Furthermore, thymol holds promise for applications in the preservatives industry, as an insect repellent, and in the perfume industry (Roufegarinejad et al., 2018; Reyhani et al., 2022; Dadé et al., 2023).

### 3.2 Antioxidant capacity of essential oils

EOs are integral aromatic constituents found in herbs and spices, conferring them with a range of biological activities, including antimicrobial, antifungal, antioxidant, and anti-inflammatory effects (Valdivieso-Ugarte et al., 2019). However, the composition of EOs in these herbs is intricate, lacking a straightforward and precise method for a comprehensive and objective evaluation of the antioxidant capacity of traditional Chinese medicines. Consequently, a variety of antioxidant assays are necessary to profile the total antioxidant potential of natural extracts in this context.

**Figure 2:**
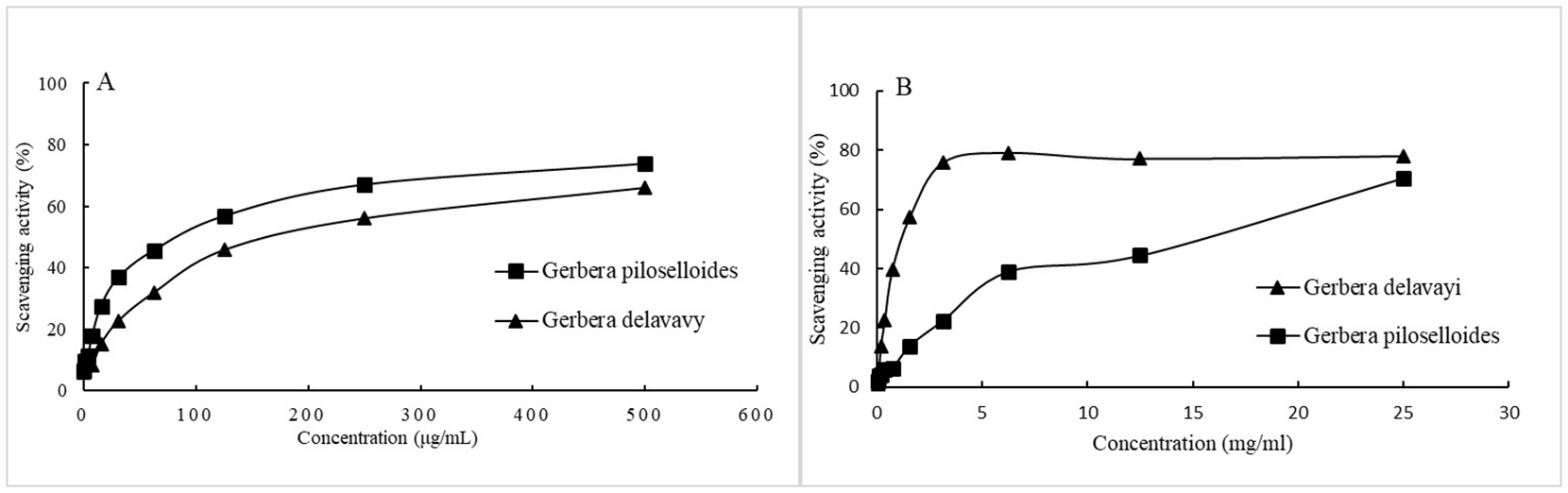
Radical scavenging activity of the essential oils from *Gerbera piloselloides* and *Gerbera delavayi* against the ABTS radical **(A)** and DPPH **(B)**. Data are represented as mean standard deviation (SD) of triplicate experiments.

We assessed the antioxidant potential of EOgp and EOgd by evaluating their efficacy in scavenging the stable free radicals ABTS and DPPH. The radical scavenging activities of the EOs are depicted in **Figures 2A** and **2B**, respectively. The concentrations of the EOs required to inhibit each radical by 50% (IC_50_) are presented in **Table 4**. Notably, *G. delavayi* exhibited a significantly higher DPPH free radical scavenging ability (IC_50_ 0.7 mg/mL) compared to *G. piloselloides* (IC_50_ 69.5 mg/mL). However, both *G. piloselloides* and *G. delavayi* demonstrated similar ABTS free radical scavenging activity, with IC_50_ values of 81 µg/mL and 105.8 µg/mL, respectively. It is documented that certain compounds with ABTS scavenging capability may not exhibit DPPH scavenging activity, which could account for the observed results (Borah et al., 2019).

**Table 4:** Antioxidant activities of essential oils from *Gerbera piloselloides* and *Gerbera delavayi*.

Sample	TPC (mg eq gallic acid/mL oil)	FRAP (mg eq vitaminC/mL oil)	IC_50_ values	
			DPPH (mg/mL)	ABTS (μg/mL)
*Gerbera piloselloides*	27.4 ± 2^*^	7.8 ± 1^*^	69.5 ± 1^***^	81 ± 5^*^
*Gerbera delavayi*	68.6 ± 4^*^	19.7 ± 5^*^	0.7 ± 0.002^***^	105.8 ± 11^**^
Vitamin C	N.T.	N.T.	0.003 ± 0.0001^***^	1.5 ± 0.02^***^

IC50, Inhibitory Concentration at 50%. Values are mean ± standard deviation (n = 3). Statistically significant differences: ^*^ *p* < 0.05, ^**^ *p* < 0.01, ^***^ *p* < 0.001. N.T., Not Tested.

**Figure 3:**
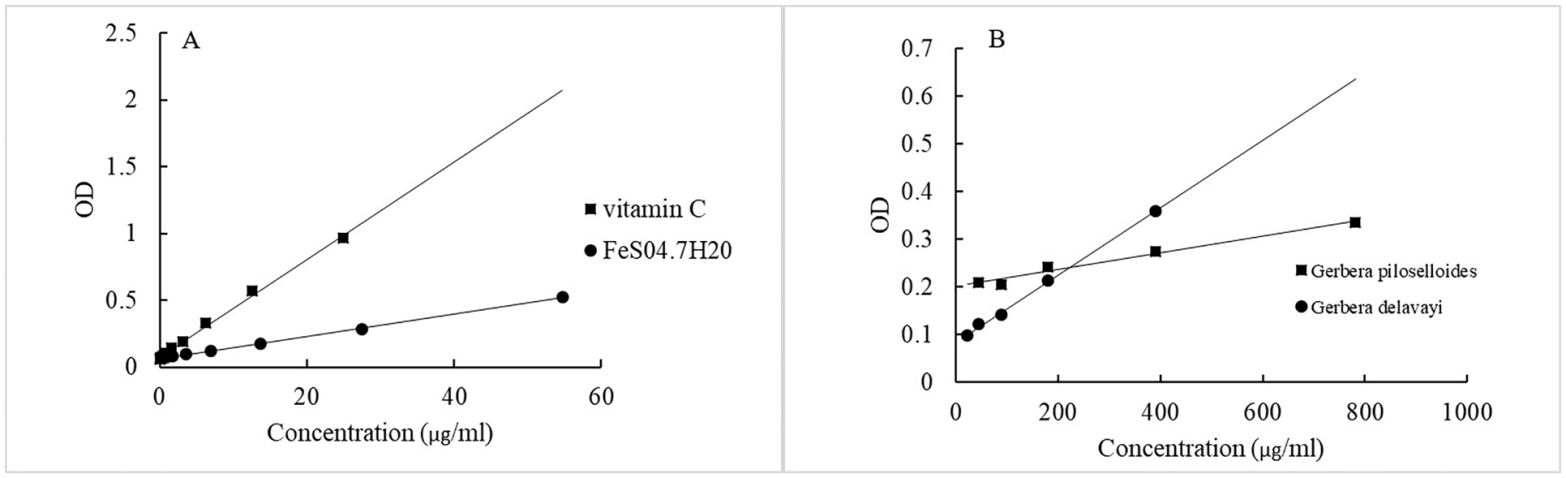
Concentration-dependent effects of antioxidants on the inhibition of the FRAP assay. **(A)** shows the correlation coefficients (r²) for vitamin C (r² = 0.996) and FeSO_4_·7H_2_O (r² = 0.999); **(B)** depicts the correlation coefficients for *Gerbera piloselloides* (r² = 0.978) and FeSO_4_·7H_2_O (r² = 0.998).

The reducing capability of the extracts was determined using a microplate reader to track the conversion of Fe^3+^ to Fe^2+^ in the presence of the extracts. An increase in absorbance is indicative of the extract’s reducing power. The influence of antioxidant concentration on FRAP inhibition is summarized in **Figure 3**. FRAP results were expressed as milligrams of vitamin C equivalent per milliliter of oil, and TPC data are provided in **Table 4**. *G. delavayi* displayed a superior antioxidant capacity, with 19.7 mg eq vitamin C/mL oil, compared to *G. piloselloides* (7.8 mg eq vitamin C/mL oil). The FRAP antioxidant activities were directly proportional to the TPC, with *G. piloselloides* and *G. delavayi* exhibiting TPC values of 27.4 mg eq gallic acid/mL oil and 68.6 mg eq gallic acid/mL oil, respectively. Studies by other researchers have also linked the antioxidant activity of *Solanum elaeagnifolium* to its TPC (Bouslamti et al., 2022). These findings suggest that TPC compounds contribute significantly to the antioxidant activity of *G. delavayi*.

### 3.3 Antimicrobial activity of essential oils

Bacterial infections continue to be a leading cause of mortality worldwide, a situation exacerbated by the persistent emergence of antibiotic resistance (Huemer et al., 2020). EO components derived from medicinal plants are noted for their high biological activity, and the quest for alternative antimicrobial agents to replace antibiotics has become a focal point of contemporary research (Coimbra et al., 2022).

This study assessed the antimicrobial potential of *G. piloselloides* and *G. delavayi* by evaluating their inhibitory effects against *Listeria*, *S. aureus*, *Salmonella*, *P. multocida*, and *E. coli*. The minimum inhibitory concentrations (MICs) of the EOs against these microbial strains are detailed in **Table 5**. The data reveal that EOgp demonstrated inhibitory activity against *Listeria* ATCC 19111, *S. aureus* CMCC26003, *Salmonella* CVCC541, *P. multocida* C48-1, and *E. coli* CVCC10141 with MICs of 6.3 mg/mL, 12.5 mg/mL, 12.5 mg/mL, 6.3 mg/mL, and 12.5 mg/mL, respectively. Similarly, Eogd exhibited efficacy against the same pathogens with MIC values of 6.3 mg/mL, 12.5 mg/mL, 12.5 mg/mL, 6.3 mg/mL, and 6.3 mg/mL, respectively. Notably, the antimicrobial potency of both EOs against *Listeria* surpassed that of chloramphenicol.

**Table 5:** Minimum Inhibitory Concentrations (MICs) of essential oils from *Gerbera piloselloides* and *Gerbera delavayi* Franch, and Chloramphenicol against selected strains.

Microbial Strains	MIC (mg/mL) for EO from *Gerbera piloselloides*	MIC (mg/mL) for EO from *Gerbera delavayi*	MIC (mg/mL) for Chloramphenicol
*S.aureus* CMCC26003	12.5	12.5	5
*Listeria* ATCC 19111	6.3	6.3	10
*Salmonella* CVCC541	12.5	12.5	5
*Pasteurella multocida* C48-1	6.3	12.5	5
*E. coli* CVCC10141	12.5	12.5	10

The biological effects of EOs are a consequence of the synergistic interaction of all molecules within the oil, and it is erroneous to attribute these effects to a single compound (Melo et al., 2020). The predominant components identified in both plant EOs were terpenes, natural products that serve diverse roles in various organisms and exhibit a wide array of structural diversity. *Listeria* has long been implicated as a primary agent of foodborne diseases in humans and animals. Cho et al. (2020) reported on the combined activities of gaseous oregano and thyme thymol EOs against *Listeria* monocytogenes. In the study by Said et al. (2016), oxygenated terpenes such as chamazulene-a degradation product, β-thujone, and camphor were identified as the main components of bioactive oils with antibacterial activity against *Listeria* monocytogenes. In the present manuscript, we also observed a high terpenoid content in both EOgp and EOgd, which may be responsible for their significant inhibitory effects against *Listeria*. The presence of these oxygenated terpenes in our extracts aligns with the findings of Said et al. (2016), suggesting that these compounds could be key contributors to the antibacterial properties observed. Our data further support the potential of natural plant-derived EOs as agents for controlling *Listeria* monocytogenes in antibacterial applications. However, it is important to note that while these EOs show promise, their safety profile must be thoroughly investigated before they can be considered for practical use.

## 4 Conclusion

In the present investigation, we assessed the chemical constituents, as well as the antioxidant and antimicrobial properties, of EOs extracted from the whole plants of *G. piloselloides* (EOgp) and *G. delavayi* (EOgd) via hydrodistillation. Our findings reveal that both EOgd and EOgp exhibit significant antioxidant capabilities and demonstrate differential inhibitory effects against five tested microbial strains. Notably, both essential oils exerted potent antibacterial effects against *Listeria* monocytogenes *in vitro*. These findings contribute to the growing body of evidence supporting the potential of these species as natural sources for the development of therapeutic products. Further research is needed to explore the specific mechanisms of action and safety profiles of these essential oils.

## Data Availability

The original contributions presented in the study are included in the article/supplementary material. Further inquiries can be directed to the corresponding author/s.

## References

[B1] Balewski Ł. Szulta S. Jalińska A. Kornicka A. (2021). Recent advances in coumarin-metal complexes with biological properties. Front. Chem. 9. doi: 10.3389/fchem.2021.781779 PMC8671816 34926402

[B2] Baran A. Kwiatkowska A. Potocki L. (2023). Antibiotics and bacterial resistance-A short story of an endless arms race. Int. J. Mol. Sci. 24, 5777. doi: 10.3390/ijms24065777 36982857 PMC10056106

[B3] Borah A. Paw M. Gogoi R. Loying R. Sarma N. Munda S.. (2019). Chemical composition, antioxidant, anti-inflammatory, anti-microbial and *in-vitro* cytotoxic efficacy of essential oil of *Curcuma caesia* Roxb. leaves: An endangered medicinal plant of North East India. Ind. Crops Prod. 129, 448–454. doi: 10.1016/j.indcrop.2018.12.035

[B4] Bouarab Chibane L. Degraeve P. Ferhout H. Bouajila J. Oulahal N. (2019). Plant antimicrobial polyphenols as potential natural food preservatives. J. Sci. Food Agric. 99, 1457–1474. doi: 10.1002/jsfa.9357 30206947

[B5] Bouslamti M. El Barnossi A. Kara M. Alotaibi B. S. Al Kamaly O. Assouguem A.. (2022). Total polyphenols content, antioxidant and antimicrobial activities of leaves of *Solanum elaeagnifolium* Cav. from Morocco. Molecules 27, 4322. doi: 10.3390/molecules27134322 35807566 PMC9268098

[B6] Cho Y. Kim H. Beuchat L. R. Ryu J. H. (2020). Synergistic activities of gaseous oregano and thyme thymol essential oils against *Listeria* monocytogenes on surfaces of a laboratory medium and radish sprouts. Food Microbiol. 86, 103357. doi: 10.1016/j.fm.2019.103357 31703857

[B7] Citarella A. Vittorio S. Dank C. Ielo L. (2024). Syntheses, reactivity, and biological applications of coumarins. Front. Chem. 12. doi: 10.3389/fchem.2024.1362992 PMC10909861 38440776

[B8] CLSI (2017). “Performance standards for antifungal susceptibility testing of yeasts,” in CLSI Supplement M60, 1 th (Clinical Laboratory Standards Institute, Wayne, PA).

[B9] CLSI (2018). “Performance standards for antimicrobial susceptibility testing,” in CLSI Supplement M100, 28 th (Clinical Laboratory Standards Institute, Wayne, PA).

[B10] Coelho M. G. da Silva A. P. de Toledo A. F. Cezar A. M. Tomaluski C. R. Barboza R. D. F.. (2023). Essential oil blend supplementation in the milk replacer of dairy calves: Performance and health. PloS One 18, e0291038. doi: 10.1371/journal.pone.0291038 37788273 PMC10547158

[B11] Coimbra A. Ferreira S. Duarte A. P. (2022). Biological properties of *Thymus zygis* essential oil with emphasis on antimicrobial activity and food application. Food Chem. 393, 133370. doi: 10.1016/j.foodchem.2022.133370 35667177

[B12] Cui H. Pan H. W. Wang P. H. Yang X. D. Zhai W. C. Dong Y.. (2018). Essential oils from *Carex meyeriana* Kunth: Optimization of hydrodistillation extraction by response surface methodology and evaluation of its antioxidant and antimicrobial activities. Ind. Crops Prod. 124, 669–676. doi: 10.1016/j.indcrop.2018.08.041

[B13] Dadé M. M. Daniele M. Reyes-Novelo E. Rodriguez-Vivas R. I. (2023). Lethal and repellent effect of amitraz, eugenol and thymol against Triatoma infestans, the main vector of *Trypanosoma cruzi* in the southern of America. Med. Vet. Entomol. 37, 574–580. doi: 10.1111/mve.12655 37052250

[B14] Hu Q. P. Cao X. M. Hao D. L. Zhang L. L. (2017). Chemical composition, antioxidant, DNA damage protective, cytotoxic and antibacterial activities of *Cyperus rotundus* rhizomes essential oil against foodborne pathogens. Sci. Rep. 7, 45231. doi: 10.1038/srep45231 28338066 PMC5364420

[B15] Huemer M. Mairpady Shambat S. Brugger S. D. Zinkernagel A. S. (2020). Antibiotic resistance and persistence-Implications for human health and treatment perspectives. EMBO Rep. 21, e51034. doi: 10.15252/embr.202051034 33400359 PMC7726816

[B16] Kıvrak İ. (2014). Analytical methods applied to assess chemical composition, nutritional value and *in vitro* bioactivities of *Terfezia olbiensis* and *Terfezia claveryi* from Turkey. Food Anal. Methods 8, 1279–1293. doi: 10.1007/s12161-014-0009-2

[B17] Larrazabal-Fuentes M. Palma J. Paredes A. Mercado A. Neira I. Lizama C.. (2019). Chemical composition, antioxidant capacity, toxicity and antibacterial activity of the essential oils from *Acantholippia deserticola* (Phil.) Moldenke (Rica rica) and *Artemisia copa* Phil. (Copa copa) extracted by microwave-assisted hydrodistillation. Ind. Crops Prod. 142, 111830. doi: 10.1016/j.indcrop.2019.111830

[B18] Liu S. Z. Feng J. Q. Wu J. Zhao W. M. (2010). A new monoterpene–coumarin and a new monoterpene–chromone from *Gerbera delavayi* . Verlag Helv. Chim. Acta 93, 2026–2029. doi: 10.1002/hlca.201000017

[B19] Liu C. He Y. Zhou K. Wang H. Zhou M. Sun J.. (2024). Mitigation of allergic asthma in mice: A compound mixture comprising luteolin, arbutin, and marmesin from *Gerbera Piloselloides* Herba by suppression of PI3K/Akt pathway. Heliyon 10, e37632. doi: 10.1016/j.heliyon.2024.e37632 39381113 PMC11456855

[B20] Liu Y. Ren H. Li K. (2024). *Litsea cubeba* essential oil: Extraction, chemical composition, antioxidant and antimicrobial properties, and applications in the food industry. J. Food Sci. 89, 4583–4603. doi: 10.1111/1750-3841.17236 39013008

[B21] Luo L. Deng J. M. Liao H. W. (2013). Analysis of volatile oil from *Gerbera piloselloides* by GC-MS. J. Chin. Medicinal Materials 36, 944–945. doi: 10.13863/j.issn1001-4454.2013.06.030 24380282

[B22] Melo C. R. Oliveira B. M. S. Santos A. C. C. Silva J. E. Ribeiro G. T. Blank A. F.. (2020). Synergistic effect of aromatic plant essential oils on the ant *Acromyrmex balzani* (Hymenoptera: Formicidae) and antifungal activity on its symbiotic fungus *Leucoagaricus gongylophorus* (Agaricales: Agaricaceae). Environ. Sci. Pollut. Res. Int. 27, 17303–17313. doi: 10.1007/s11356-020-08170-z 32157534

[B23] Oliveira de Veras B. Melo de Oliveira M. B. Granja da Silva Oliveira F. Queiroz Dos Santos Y. Saturnino de Oliveira J. R. Lucia de Menezes Lima V.. (2020). Chemical composition and evaluation of the antinociceptive, antioxidant and antimicrobial effects of essential oil from *Hymenaea cangaceira* (Pinto, Mansano & Azevedo) native to Brazil: A natural medicine. J. Ethnopharmacol. 247, 112265. doi: 10.1016/j.jep.2019.112265 31580941

[B24] Petric D. Mravcakova D. Kuckova K. Cobanova K. Kisidayova S. Cieslak A.. (2020). Effect of dry medicinal plants (wormwood, chamomile, fumitory and mallow) on *in vitro* ruminal antioxidant capacity and fermentation patterns of sheep. J. Anim. Physiol. Anim. Nutr. (Berl.) 104, 1219–1232. doi: 10.1111/jpn.13349 32202350

[B25] Rehman A. Jafari S. M. Aadil R. M. Assadpour E. Randhawa M. A. Mahmood S. (2020). Development of active food packaging via incorporation of biopolymeric nanocarriers containing essential oils. Trends Food Sci. Technol. 101, 106–121. doi: 10.1016/j.tifs.2020.05.001

[B26] Reyhani Y. Iranshahi M. Taghizadeh S. F. Saberi S. Farhadi F. (2022). An evaluation of qH NMR: A complementary approach to GC-FID for quantification of Thymol and trans-Anethole in essential oils and supplements. J. Pharm. Biomed. Anal. 220, 114992. doi: 10.1016/j.jpba.2022.114992 35985134

[B27] Roufegarinejad L. Jahanban-Esfahlan A. Sajed-Amin S. Panahi-Azar V. Tabibiazar M. (2018). Molecular interactions of thymol with bovine serum albumin: Spectroscopic and molecular docking studies. J. Mol. Recognit. 31, e2704. doi: 10.1002/jmr.2704 29600590

[B28] Said M.-A. Militello M. Saia S. Settanni L. Aleo A. Mammina C.. (2016). *Artemisia arborescens* essential oil composition, enantiomeric distribution, and antimicrobial activity from different wild populations from the mediterranean area. Chem. Biodivers. 13, 1095–1102. doi: 10.1002/cbdv.201500510 27447740

[B29] Semerdjieva I. B. Shiwakoti S. Cantrell C. L. Zheljazkov V. D. Astatkie T. Schlegel V.. (2019). Hydrodistillation extraction kinetics regression models for essential oil yield and composition in *Juniperus virginiana*, *J. excelsa*, and *J. sabina* . Molecules 24, 986. doi: 10.3390/molecules24050986 30862073 PMC6429388

[B30] Skała E. Rijo P. Garcia C. Sitarek P. Kalemba D. Toma M.. (2016). The essential oils of *Rhaponticum carthamoides* hairy roots and roots of woil-grown plants: Chemical composition and antimicrobial, anti-inflammatory, and antioxidant activities. Oxid. Med. Cell Longev. 2016, 8505384. doi: 10.1155/2016/8505384 28074117 PMC5203915

[B31] Szöke E. Máday E. Marczal G. Lemberkovics E. (2004). Analysis of biologically active essential oil components of chamomiles in Hungary (*in vivo* - *in vitro*). Acta Hortic. 597, 275–284. doi: 10.17660/ActaHortic.2003.597.40

[B32] Taherpour A. A. Maroofi H. Bajelani O. Larijani K. (2010). Chemical composition of the essential oil of *Valeriana alliariifolia* Adams of Iran. Nat. Prod. Res. 24, 973–978. doi: 10.1080/14786410902900010 20496237

[B33] Tang X. J. Zhang Y. Huang H. R. Fang T. Z. Xu S. B. (2003). Analysis and comparison of the essential oil from the leaves, caudexes and roots of *Gerbera piloselloides* cass. Acta Scientiarum Naturalium Universitatis Sunyatseni 42, 125.

[B34] Thabet A. A. Moghannem S. Ayoub I. M. Youssef F. S. Al Sayed E. Singab A. N. B. (2022). GC/MS profiling of essential oils from *Bontia daphnoides* L., chemometric discrimination, isolation of dehydroepingaione and evaluation of antiviral activity. Sci. Rep. 12, 17707. doi: 10.1038/s41598-022-22174-4 36271233 PMC9587025

[B35] Tian H. Zhao H. Zhou H. Zhang Y. (2019). Chemical composition and antimicrobial activity of the essential oil from the aerial part of *Dictamnus dasycarpus* Turcz. Ind. Crops Prod. 140, 111713. doi: 10.1016/j.indcrop.2019.111713

[B36] Valdivieso-Ugarte M. Gomez-Llorente C. Plaza-Díaz J. Gil Á. (2019). Antimicrobial, antioxidant, and immunomodulatory properties of essential oils: A systematic review. Nutrients 11, 2786. doi: 10.3390/nu11112786 31731683 PMC6893664

[B37] Wang J. Petrova V. Wu S. B. Zhu M. Kennelly E. J. Long C. (2014). Antioxidants from *Gerbera piloselloides*: an ethnomedicinal plant from southwestern China. Nat. Prod. Res. 28, 2072–2075. doi: 10.1080/14786419.2014.924000 24895993

[B38] Wu Z. Y. Raven P. H. Hong D. Y. (2005). Flora of China. Vol 14: Apiaceae through Ericaceae (Beijing, China: Science Press), 451–471.

[B39] Wu H. Zhao F. Li Q. Huang J. Ju J. (2024). Antifungal mechanism of essential oil against foodborne fungi and its application in the preservation of baked food. Crit. Rev. Food Sci. Nutr. 64, 2695–2707. doi: 10.1080/10408398.2022.2124950 36129051

[B40] Xu X. D. Zheng W. Chen L. Q. Wen J. (2017). Genetic diversity and population structure of *Gerbera delavayi* (Asteraceae) in Southwest China: Implications for conservation. Ann. Bot. Fenn. 54, 409–422. doi: 10.5735/085.054.0623

[B41] Zengin G. Atasagun B. Zakariyyah Aumeeruddy M. Saleem H. Mollica A. Babak Bahadori M.. (2019). Phenolic profiling and *in vitro* biological properties of two Lamiaceae species (*Salvia modesta* and *Thymus argaeus*): A comprehensive evaluation. Ind. Crops Prod. 128, 308–314. doi: 10.1016/j.indcrop.2018.11.027

[B42] Zhao C. Gao H. Li J. Yu M. Wu J. Zhang H.. (2022). Bioactive constituents from *Gerbera piloselloides* with anti-inflammatory and antiproliferative activities. Fitoterapia 161, 105258. doi: 10.1016/j.fitote.2022.105258 35901976

[B43] Zhao C. Li J. Hu Y. Li L. Yu M. Huang Y.. (2024). (+)/(-)-Gerbeloid A, a pair of unprecedented coumarin-based polycyclic meroterpenoid enantiomers from *Gerbera piloselloides*: Structural elucidation, semi-synthesis, and lipid-lowering activity. Acta Pharm. Sin. B 14, 2657–2668. doi: 10.1016/j.apsb.2024.03.035 38828137 PMC11143508

[B44] Zheng W. Xu X. Wen J. (2017). The ethnic textile use of natural fibers from fireweed (*Gerbera delavayi*) in Southwest China. Econ. Bot. 71, 380–386. doi: 10.1007/s12231-017-9394-y

[B45] Zhou K. Lu D. You J. Liu T. Sun J. Lu Y.. (2022). Integrated plasma pharmacochemistry and network pharmacology to explore the mechanism of *Gerberae Piloselloidis* Herba in treatment of allergic asthma. J. Ethnopharmacol. 298, 115624. doi: 10.1016/j.jep.2022.115624 35970314

[B46] Zhou W. Wang Z. Mo H. Zhao Y. Li H. Zhang H.. (2019). Thymol mediates bactericidal activity against *Staphylococcus aureus* by targeting an Aldo-Keto reductase and consequent depletion of NADPH. J. Agric. Food Chem. 67, 8382–8392. doi: 10.1021/acs.jafc.9b03517 31271032

